# Highly diverse chromoviruses of *Beta vulgaris* are classified by chromodomains and chromosomal integration

**DOI:** 10.1186/1759-8753-4-8

**Published:** 2013-03-01

**Authors:** Beatrice Weber, Tony Heitkam, Daniela Holtgräwe, Bernd Weisshaar, André E Minoche, Juliane C Dohm, Heinz Himmelbauer, Thomas Schmidt

**Affiliations:** 1 Institute of Botany, Dresden University of Technology, Dresden D-01062, Germany; 2 Center for Biotechnology, University of Bielefeld, Bielefeld, D-33594, Germany; 3 Centre for Genomic Regulation (CRG) and UPF, Barcelona, E-08003, Spain; 4 Max Planck Institute for Molecular Genetics, Ihnestr. 63-73, Berlin, D-14195, Germany

**Keywords:** Ty3-*gypsy* retrotransposon, Chromovirus, CR, Chromodomain, Heterochromatin, Beta vulgaris

## Abstract

**Background:**

Chromoviruses are one of the three genera of Ty3-*gypsy* long terminal repeat (LTR) retrotransposons, and are present in high copy numbers in plant genomes. They are widely distributed within the plant kingdom, with representatives even in lower plants such as green and red algae. Their hallmark is the presence of a chromodomain at the C-terminus of the integrase. The chromodomain exhibits structural characteristics similar to proteins of the heterochromatin protein 1 (HP1) family, which mediate the binding of each chromovirus type to specific histone variants. A specific integration via the chromodomain has been shown for only a few chromoviruses. However, a detailed study of different chromoviral clades populating a single plant genome has not yet been carried out.

**Results:**

We conducted a comprehensive survey of chromoviruses within the *Beta vulgaris* (sugar beet) genome, and found a highly diverse chromovirus population, with significant differences in element size, primarily caused by their flanking LTRs. In total, we identified and annotated full-length members of 16 families belonging to the four plant chromoviral clades: CRM, Tekay, Reina, and Galadriel. The families within each clade are structurally highly conserved; in particular, the position of the chromodomain coding region relative to the polypurine tract is clade-specific. Two distinct groups of chromodomains were identified. The group II chromodomain was present in three chromoviral clades, whereas families of the CRM clade contained a more divergent motif. Physical mapping using representatives of all four clades identified a clade-specific integration pattern. For some chromoviral families, we detected the presence of expressed sequence tags, indicating transcriptional activity.

**Conclusions:**

We present a detailed study of chromoviruses, belonging to the four major clades, which populate a single plant genome. Our results illustrate the diversity and family structure of *B. vulgaris* chromoviruses, and emphasize the role of chromodomains in the targeted integration of these viruses. We suggest that the diverse sets of plant chromoviruses with their different localization patterns might help to facilitate plant-genome organization in a structural and functional manner.

## Background

In plants, long terminal repeat (LTR) retrotransposons constitute the most abundant class of transposable elements (TEs), and represent more than 50% of the genome in some species [1,2]. Their massive amplification and dynamic nature play a central role in the organization, function, and evolution of plant genomes [3-5]. The ‘copy and paste’ propagation by reverse transcription of RNA intermediates can rapidly increase the copy number of LTR retrotransposons and, hence, the genome size [6,7]. However, the excision of LTR retrotransposons through diverse recombination processes counteracts genome expansion, and thus contributes to the dynamic balance of host genome size [8,9].

The LTRs flanking the coding region contain promoter motifs that drive transcription. The proteins essential for the reverse transcription and integration of a new LTR retrotransposon copy are encoded as a *gag-pol* polyprotein organized in one or two open reading frames (ORFs). Based on gene arrangement, plant LTR retrotransposons can be further sub-classified into the Ty1-*copia* order (*Pseudoviridae*) and the Ty3-*gypsy* order (*Metaviridae*), with the latter having the same domain arrangement as retroviruses (*Orthoviridae*) [10,11].

The physical mapping of LTR retrotransposons by fluorescence *in situ* hybridization (FISH) and the accessibility of complete plant-genome sequences have yielded considerable information on their chromosomal and genomic organization. Thereby, a non-random genomic distribution with accumulation in particular chromosomal regions is apparent, indicating different target specificities [12]. Several studies have identified a role for the integrase in different patterns of retrotransposon integration [13,14]. A specific lineage of Ty3-*gypsy* retrotransposons is characterized by the presence of a chromodomain (*chr*omatin *o*rganization *mo*difier domain) at the C-terminal region of the integrase, and members of this lineage are referred to as chromoviruses [15,16]. Proteins containing chromodomains are strongly related to chromatin modification and gene regulation [17], and are able to interact with proteins, RNA, and DNA [18]. Recent studies have shown that the chromodomain specifies the target site preference of LTR retrotransposons by the recognition of characteristic chromatin modifications [19,20]. Chromoviruses represent the ancient and most widespread lineage of Ty3-*gypsy* retrotransposons, and the four chromoviral clades, Tekay, CRM, Galadriel, and Reina, are widely distributed throughout gymnosperms and angiosperms [16].

Recently, the chromosomal and genomic organization of centromere-specific chromoviruses in the wild beet genus *Patellifolia*, which is closely related to the beet genus *Beta*, were described [21]. The genus *Beta* belongs to the family Amaranthaceae and is subdivided into the sections *Beta*, *Corollinae*, and *Nanae*. All cultivated species (sugar beet, fodder beet, garden beet, and chard) belong exclusively to the section *Beta*[22]. The *Beta vulgaris* (sugar beet) genome is 758 Mb in size [23] and contains at least 63% repetitive sequences [24]. The access to early assembly versions for a draft of the *B. vulgaris* genome sequence enabled bioinformatic identification of complete chromoviruses. These data have facilitated large-scale studies of the structure, variability, and evolution of LTR retrotransposons in the genus *Beta* to complement and extend the knowledge of the repetitive DNA fraction [21,25,26].

In this study, we characterize all four chromoviral clades populating the genome of *B. vulgaris*. Based on the amino acid composition of their chromodomain, all *B. vulgaris* chromoviruses can be attributed to one of two distinct groups. We investigated the evolution of the chromoviral clades within the beet genera *Beta* and *Patellifolia*, and found that the chromoviruses are highly divergent. Their chromosomal distribution patterns might result from a targeted integration mediated by different chromodomains.

## Results

### Structure of the chromoviruses in *Beta vulgaris*

To analyze *B. vulgaris* chromoviral retrotransposons, we conducted *tBLASTn* searches against contigs of the *B. vulgaris* genome sequence draft, using domains of the *gag-pol* polyproteins of the chromoviral Tekay, CRM, Galadriel, and Reina clades.

We selected 65 contigs with an e-value of less than e-100 to clearly separate these hits from other Ty3-*gypsy* retrotransposons, and a sequence length of more than 6.5 kb to increase the detection of full-length elements. In order to select structurally intact and complete chromoviruses, both undisrupted internal domains and the presence of target site duplications (TSDs) were used as criteria. In total, 20 full-length chromoviruses were detected, which are bordered by TSDs of 5 bp in length, and belong to the Tekay, CRM, and Reina clades (Table 1). The remaining 45 contigs harbored incomplete, recombined, or highly degenerated chromoviral retrotransposons.

**Table 1:** **Chromoviruses of *Beta vulgaris***

**Clade**	**Name**	**Total length, bp**	**LTR length 5**^**′**^**/3**^**′**^**, bp**	**TSD, bp**	**LTR divergence,%**	**Age, mya**	**RefBeet contig**
CRM	***Beetle*3**^**a**^	5917	744/742	GTA(T/A)A	2.47	0.82	402713
	*Beetle*3-1	5908	753/753	AGGAG	3.68	1.225	669577
	***Beetle*4**^**a**^	6175	886/889	TAATA	4.07	1.35	412171
	*Beetle*4-1	6182	886/890	TAATA	4.06	1.35	554084
	*Beetle*4-2	6169	885/885	CAACA	1.25	0.42	319766
	***Beetle*5**^**a**^	6654	1339^b^/679	AAC(G/T)A	1.79	0.6	483491
	*Beetle*5-1	5941	659/637	TATCA	2.88	0.96	400864
	*Beetle*5-2	5961	679/675	CAATA	3.96	1.32	22920
	***Beetle*6**^**a**^	5315	624/422^c^	TCAGG	(2.91)	(0.9)	636287
	***Beetle*7**^**a**^	6695	1086/1086	ACAA(C/A)	2.62	0.87	51293^d^
Tekay	***Bongo*1**^**a**^	10329	2669/3000^b^	AAAAT	5.23	1.75	548721
	***Bongo*2**^**a**^	8417	1049^c^/2743	TAGTA	(3.84)	(1.29)	02834
	***Bongo*3**^**a**^	11565	2561/2561	GAGCG	0.08	0.026	EF101866^e^
Reina	***Bingo*1**^**a**^	5811	431/430	TAAAT	0.23	0.08	262052
	*Bingo*1-1	5810	425/423	GATTG	1.68	0.6	382156
	***Bingo*2**^**a**^	5563	503/500	CTAAC	1.01	0.34	626667
	***Bingo*3**^**a**^	5807	453/453	GTAAG	0.67	0.22	596930
	***Bingo*4**^**a**^	5452	392/392	TGATG	0.77	0.026	429428
	***Bingo*5**^**a**^	5320	343/337	ACCAC	1.80	0.6	111648
	***Bingo*6**^**a**^	5261	293/293	CGCAA	1.03	0.345	627491
	***Bingo*7**^**a**^	5476	494/494	GGGTT	1.64	0.546	343138
Galadriel	***Beon*1**^**a**^	6038	631/631	GTAGG	0	Identical LTRs	19150^d^

^**a**^Chromoviral family.

^b^Contains insertion.

^c^Contains deletion.

^d^RefBeet 0.4.

^e^Accession number of a complete sequenced *B. vulgaris* bacterial artificial chromosome (BAC).

Members of the Galadriel clade were detectable only using a refined *tBLASTn* query based on the Galadriel integrase amino acid sequence [27]. A complete internal chromoviral sequence, harboring no stop codons or frameshifts, but lacking the LTRs, was identified, and the reverse transcriptase (RT) gene sequence was used as a probe to detect the sugar-beet bacterial artificial chromosome (BAC) 54O24 by hybridization to high-density filters [28]. The Galadriel sequence was obtained by an adaptor primed suppression PCR method [29] using the DNA of BAC 54O24 as template. The 21 retrotransposons belong to the 4 plant chromoviral clades, and were designated as *Beetle* (CRM clade), *Bingo* (Reina clade), *Bongo* (Tekay clade), and *Beon* (Galadriel clade).

Furthermore, the Ty3-*gypsy* retrotransposon SCHMIDT, previously detected in a *B. vulgaris* BAC sequence [30] and by sequence analysis of genomic clones from a *c*_*o*_*t*-1 library of *B. vulgaris*[31], represents an intact chromovirus of the Tekay clade. In accordance with the nomenclature of *Beta* chromoviruses, we assigned SCHMIDT to the *Bongo* chromoviruses, and designated it as *Bongo*3.

**Figure 1:**
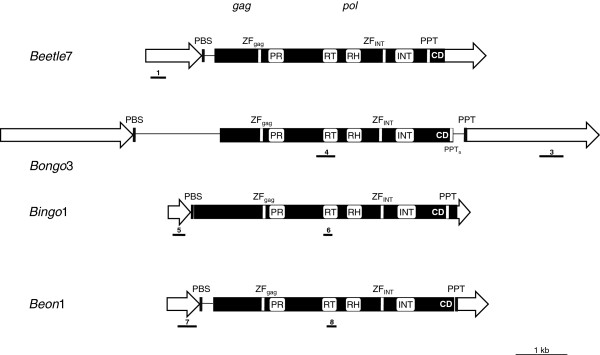
**Structural organization of representative members of *Beta vulgaris *chromoviruses. **Open arrows represent the long terminal repeats (LTRs). The primer binding site (PBS) and the polypurine tracts (PPTs) are indicated. Conserved domains are shown: *gag *and *pol*, with the catalytic domains of protease (PR), reverse transcriptase (RT), RNaseH (RH), and integrase (INT), containing the chromodomain (CD) at the C-terminus. Note that the ORF of the *gag-pol* polyprotein in *Beetle*7 and *Bingo*1 extends into the 3^′^ LTR. Zinc finger (ZF) motifs of the protease and integrase are indicated. The additional PPT of *Bongo*3 is designated PPT_a_. Numbered dashes show the position of probes used for hybridization experiments. The corresponding primers used for DNA probe amplification are listed in Table 2.

For the detailed molecular investigation, one representative of each of the four chromoviral clades was chosen (*Beetle*7, *Bongo*3, *Bingo*1, *Beon*1), based on the presence of an intact ORF. All *Beta* chromoviruses harbor a conserved *gag-pol* polyprotein with a similar length for the *pol* region and similar distances between the catalytic regions (RNA binding site/protease/reverse transcriptase/RNaseH/integrase). The retroviral genes *gag* and *pol* are encoded in a continuous single open reading frame (ORF) (Figure 1). By contrast, large size differences are seen between the families, originating from the differing LTR length (Table 1) and the 5^′^ and 3^′^ untranslated regions (UTRs). Comparing *Bingo*1 and *Bongo*3, these structural differences lead to a size ratio of 1:2, even though their deduced respective *gag-pol* polyproteins are similar in length. *Beta* chromoviruses show a typical primer binding site (PBS) complementary to the 3^′^ end of the tRNA methionine, with up to three nucleotides distance to the 5^′^ LTR, whereas the polypurine tract (PPT), consisting of 7 to 14 purines, was detected immediately upstream of the 3^′^ LTR (see Additional file 1: Figure S1).

In *Bongo*3, the putative functional PPT is located immediately upstream of the 3^′^ LTR, and an additional PPT (PPT_a_) is located downstream of the chromodomain and overlaps with the TAA stop codon of the *gag-pol* ORF (Figure 1). *Bongo*1 and *Bongo*2, as well as members of other clades, do not contain duplicated PPTs.

**Figure 2:**
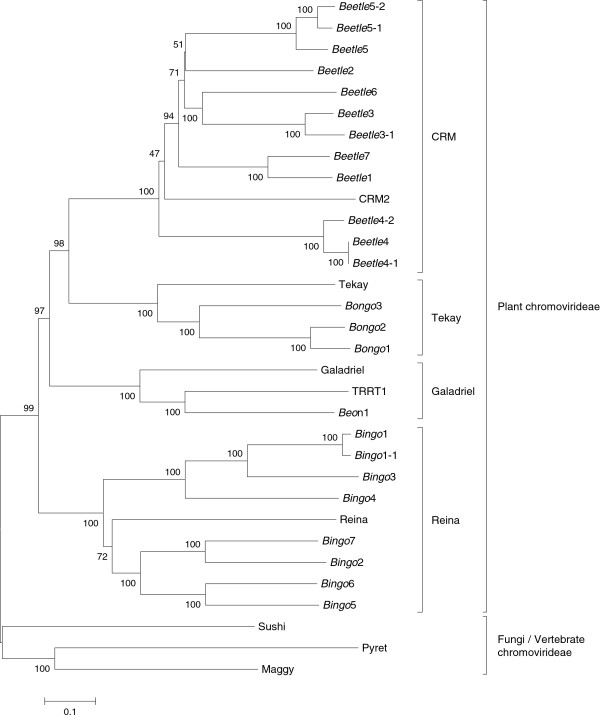
**Dendrogram showing the relationship of the *Beta vulgaris *chromoviruses to other chromodomain-containing Ty3-*gypsy *retrotransposons. **The neighbor-joining tree was constructed from the alignment of the complete *gag-pol *polyprotein sequences of *B. vulgaris* and representative chromoviruses from other plant genomes (CRM, Tekay, and Reina from *Zea mays*, Galadriel from *Lycopersicon esculentum*). For further classification, the recently described Ty3-*gypsy *retrotransposon TRRT1 (tomato ribosomal (r)DNA-related retrotransposon) was included. The fungi and vertebrate chromoviruses from *Magnaportha grisea *(Maggy) and *Takifugu rubripes* (Sushi-ichi) were included as outgroup elements to root the tree. Bootstrap values are indicated as a percentage of 1,000 replicates.

To assign the *B. vulgaris* chromoviruses to the plant chromoviral clades, a dendrogram was constructed using nucleotide sequence alignment of the complete ORF of the *gag-pol*-polyprotein (Figure 2). Supported by high bootstrap values, all *Beta* chromoviruses are assigned to one of the four plant chromoviral clades. The five *Beetle* elements (*Beetle*3 to 7) were clearly grouped into the CRM clade; the *Bongo* families (*Bongo*1 to 3) into the Tekay clade; the *Bingo* families (*Bingo*1 to 7) into the Reina clade; and *Beon*1 was assigned to the Galadriel clade. The non-plant chromoviruses Sushi-ichi from *Takifugu rubripes*[32] and Maggy and Pyret from *Magnaporthe grisea*[33,34] were placed on separate branches.

### Genome-wide analysis of chromoviral reverse transcriptases

**Figure 3:**
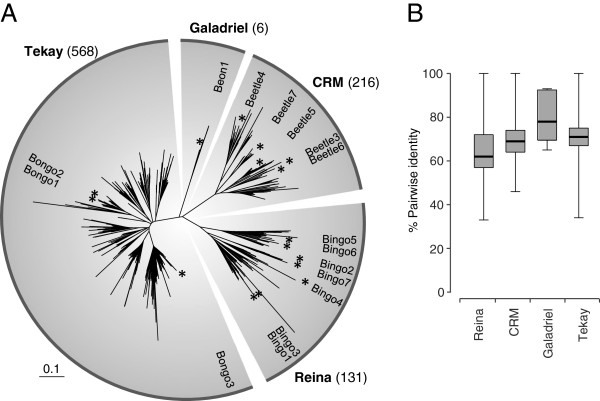
**Diversity of chromoviral lineages in *B. vulgaris*. (A) **Dendrogram representing the amino acid sequences of 921 reverse transcriptases (RTs) of chromoviral Ty3-*gypsy *retrotransposons in *B. vulgaris*. Stars represent the position of chromoviral families (Tekay clade: *Bongo*1 to 3; Galadriel clade: *Beon*1; CRM clade: *Beetle*3 to 7; Reina clade: *Bingo*1 to 7) analyzed in detail. **(B) **Box plot representing the amino acid sequence identity between the RTs of all four clades. The median is indicated with a vertical line, and the upper and lower quartiles are within the box.

In order to study the chromoviral diversity within the *B. vulgaris* genome, a Hidden Markov Model (HMM)-based strategy was used to identify 921 RTs of chromoviruses within the available genome sequence. Their putative amino acid sequences were aligned, and a neighbor-joining tree was constructed to visualize their relationship (Figure 3A). All RTs grouped to one of the four plant chromoviral clades. Within clades, pairwise RT sequence identities were calculated and displayed as a box plot (Figure 3B; the median is indicated by a horizontal line, with the upper and lower quartiles are within the box, while the whiskers include the minimum and maximum values). The Reina clade RTs (*Bingo* families) had the highest sequence diversity, with a low median identity (62%) and a mean identity of 65%. The *Bongo* families within the Tekay clade comprised the majority of the RTs found (n = 568). With an average identity of 72%, they are split into many families with similar members. This was also the case for the 216 *Beetle* members of the CRM clade (average identity 71%). In terms of member number and sequence identity, RTs of the Galadriel clade are set apart from all other chromoviral sequences in *B. vulgaris*, with only six highly identical RTs (average identity 81%) constituting this branch.

### Chromodomains distinguish the chromoviral clades

The position of the *gag-pol* ORF termini and the localization of the chromodomain in the integrase with reference to the PPT are the major characteristics facilitating the separation of chromoviruses into clades. Previous studies have shown that the *gag-pol* ORF of the CRM clade members extends into the 3^′^ LTR, with the chromodomain situated downstream of the PPT [21,35]. For the remaining chromoviral clades, the chromodomain is located upstream of the PPT. In the chromoviruses *Bingo*1 to 4 and *Bingo*7, an extension of the ORF into the flanking LTR was found, whereas in *Bingo*5 and *Bingo*6, the *gag-pol* ORFs were seen to terminate upstream of the 3^′^ LTR.

A comprehensive investigation of the predicted integrase amino acid sequence harboring the chromodomain was carried out. The integrase sequence could be separated into two regions (see Additional file 2; Figure S2). The 5^′^ region spans the integrase region between the ZF and the GPF/GPY motif (positions 1 to 313) and is highly conserved throughout plant chromoviruses. The typical ZF HHCC is followed by the D, D_35_E motif. There is a marked enrichment of positively charged residues upstream of the GPF/GPY motif. In particular, the *Beetle* families are enriched in the amino acids arginine (K) and lysine (R). Interestingly, the proline (P) upstream of the RK-rich region implies similarity to nuclear localization signals (NLSs) with the motif PxR/KKxK.

**Figure 4:**
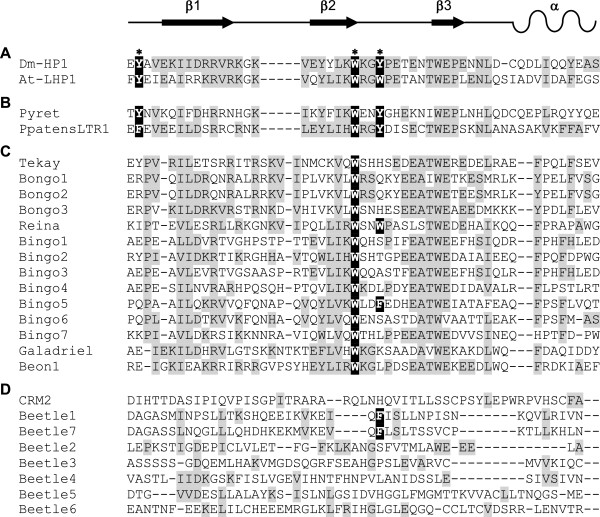
**Comparison of the classic and chromovirus-derived chromodomains. **Chromodomain structure of the heterochromatin protein 1 (HP1) family and diverse chromoviruses shown as alignment of multiple amino acid sequences. **(A) **Amino acid sequences of chromodomains of the HP1 proteins from *Drosophila melanogaster *(Dm-HP1) and *Arabidopsis thaliana *(At-LHP1), with the secondary structure illustrated at the top. The residues that constitute an aromatic cage are boxed in black and marked by asterisks. **(B) **In chromoviruses with group I chromodomains, the three conserved aromatic residues Y, W, and Y are present. **(C) **Group II chromodomain-containing chromoviruses usually lack the first and third conserved aromatic residue. **(D)** The CR motif of the centromeric *Beetle *chromoviruses is substantially diverged. Similar amino acids are shaded in grey.

The 3^′^ region of the integrase contains the chromodomain. When compared with heterochromatin protein-1 (HP1) [36,37], the chromodomains could be assigned to one of two groups (Figure 4). Members of group I show conservation in the residues tyrosine, tryptophan, and tyrosine (YWY), which in HP1 constitute an aromatic cage recognizing the methylated lysine 9 of histone 3 (H3K9me). Group II chromodomains are characterized by lack of the first and third conserved aromatic residues, and to date have only been detected in plants [19,38]. The families *Bongo* and *Bingo*, are typical representatives of chromoviruses containing a group II chromodomain. The centromeric plant chromoviruses encode a substantially diverged CR motif [39,40], and the five *Beetle* families show a much lower degree of sequence conservation within the CR motif.

### Genomic organization of *Beta* chromoviruses

**Figure 5:**
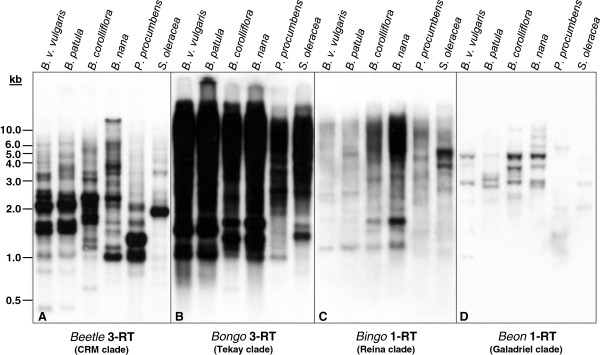
**Genomic organization and abundance of the chromoviral clades CRM, Tekay, Reina, and Galadriel in species of the genera *Beta *and *Patellifolia*. **Genomic *Hin*dIII-restricted DNA was analyzed by comparative Southern hybridization using a probe from the reverse transcriptase (RT) sequence of **(A)***Beetle*3 (CRM clade), **(B)***Bongo*3 (Tekay clade), **(C)***Bingo*1 (Reina clade), and **(D)***Beon*1 (Galadriel clade). The following species from the section *Beta* were analyzed: (1) *B. vulgaris ssp. vulgaris *KWS2320 (cultivated beet), (2) *B. patula* (wild beet). Wild beets were used from the sections *Corollinae* ((3) *B. corolliflora*), and *Nanae *((4) *B. nana*). Additionally, he species *Patellifolia procumbens *(5) from the genus *Patellifolia*, formerly classified as *Beta procumbens *of the genus *Beta* was analyzed. *Spinacia oleracea *(6) was used as outgroup species. The exposition times were 8 hours for filters A and B, and 24 hours for filters C and D.

Comparative hybridization to *Hin*dIII-restricted genomic DNA was performed to investigate abundance, genomic organization, and distribution of the four chromoviral clades CRM, Reina, Tekay, and Galadriel in the genera *Beta* (sections *Beta*, *Corollinae*, and *Nanae*) and *Patellifolia* (Figure 5; see Additional file 3). An RT probe of the clade members *Beetle*3, *Bongo*3, *Bingo*1, and *Beon*1 enabled detection of their respective families, and of related families within each clade.

The strong signals indicated the high abundance of *Beetle* and *Bongo* retrotransposons (Figure 5A, B). By contrast, *Bingo* and *Beon* chromoviruses had either lower copy numbers or more diverged members (Figure 5C, D). Signals over a wide range of molecular weights were visible for *Beetle*, *Bongo*, and *Bingo* (Figure 5A–C), but not for *Beon* chromoviruses (Figure 5D). However, the presence of strongly hybridizing fragments in all clades indicates the existence of multiple copies with conserved restriction sites across most species. A similar banding pattern was detected in the cultivated sugar beet *B. vulgaris ssp. vulgaris* (lane 1) and the wild beet *B. patula* (lane 2), both belonging to the section *Beta*. Apart from *Beetle*3 (Figure 5A), similar results were found for *B. corolliflora* (lane 3) and *B. nana* (lane 4), with conserved fragments in most of the autoradiograms, suggesting a close relationship of the various chromoviruses. Additionally, the wild beet *Patellifolia procumbens* (lane 5), formerly assigned to the genus *Beta*, and the more distant spinach (*Spinacia oleracea*) (lane 6) were analyzed as outgroups. The genomes of both species also contain chromoviruses of the clades CRM, Tekay, Galadriel and Reina.

The Southern blotting experiments indicate the widespread distribution of chromoviruses within the order Amaranthaceae. The differences in copy number are clade-specific rather than species-specific. The *Bongo* families of the Tekay clade showed the highest amplification, whereas Galadriel clade members were much less abundant throughout all analyzed species. This is consistent with the genome-wide analysis of RT sequences of *B. vulgaris*, for which more than 60% of the RTs were found to belong to the Tekay clade, followed by 23% and 14% for the CRM and Reina clades, respectively.

### Estimation of the transposition time

To estimate the transposition time of individual chromoviral copies, we used the divergence of the LTR sequences, as these pairs are usually identical upon integration [2]. Assessing the 22 full-length chromoviruses, we found that elements of the Reina clade had inserted most recently into the *B. vulgaris* genome, with an average transposition time of 0.35 Mya. Similarly, the *Bingo* families transposed between 26,000 and 600,000 years ago (Table 1). The average transposition times estimated for the CRM clade families and the Tekay chromoviruses were approximately equal (one million years ago). For the estimation of the transposition time of *Beetle*6 and *Bongo*2, which contain one partially deleted LTR, the matching LTR regions were used, resulting in respective timeframes of 0.97 and 1.29 million years ago.

### Chromosomal localization of chromoviruses

**Figure 6:**
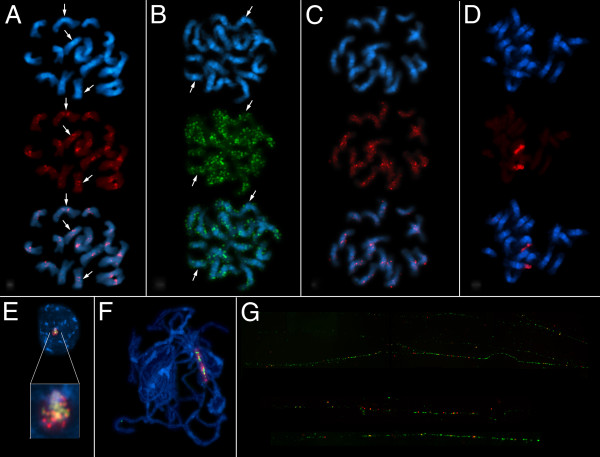
**Physical mapping of *Beta vulgaris *chromoviruses. **Localization of different chromoviruses on metaphase chromosomes and interphase nucleus of *B. vulgaris *by fluorescence *in situ* hybridization (FISH) using long terminal repeat (LTR)-specific probes. High-resolution FISH was performed on pachytene chromosomes and DNA fibers. In each panel, the stained (4^′^,6^′^-diamidino-2-phenylindole; DAPI: blue fluorescence) DNA shows the morphology of the chromosomes. Hybridized probes were detected with cyanine 3 (Cy3; red fluorescence) and fluorescein isothiocyanate (FITC; green fluorescence). **(A) **The LTR of *Beetle*7 hybridized to centromeric and peri centromeric heterochromatin of most chromosomes; examples for depletion from centromeric heterochromatin are marked by arrows. **(B) **The hybridization with the LTR probe of *Bongo*3 visualized the clustered organization of the *Bongo*3 family along all chromosomes. Reduced hybridization to some centromeric regions was detectable (examples marked by arrows). **(C)** Amplification of *Bingo*1 chromoviruses was seen as strong signals largely in the peri-centromeric regions. **(D) **The LTR of *Beon*1 (red fluorescence) hybridized only to chromosome 1, harboring the ribosomal (r)RNA genes. **(E-G) **The localization of *Beon*1 within the rDNA locus was confirmed by multi-color FISH analysis using a probe for the 18S rRNA (green fluorescence). **(E) **The signals of both DNA probes overlapped at the rDNA of the interphase nuclei. **(F) **FISH to chromosomes of *B. vulgaris *at the pachytene stage of meiosis showed interspersion of 18 rRNA genes with *Beon*1 copies. **(G) **The hybridization to extended DNA fibers revealed the physical order of *Beon*1 copies within the nucleolar organizer region (NOR) , where not every rDNA array was found to harbor a *Beon*1 element.

The physical organization of the chromovirus families was investigated by FISH on *B. vulgaris* chromosomes (Figure 6). We used LTR probes specific for each family to prevent cross-hybridization with unrelated retrotransposons.

The LTR probe of *Beetle*7 hybridized to the centromeric and peri-centromeric heterochromatin of most chromosomes (Figure 6A).

The FISH image of *Bongo*3 showed a pattern of widely dispersed signals along the length of all chromosome arms, including the intercalary and centromeric heterochromatin of both arms (Figure 6B); however, reduced hybridization to some centromeric regions was seen (Figure 6B, arrows). Strong signals were detected on both chromatids, indicating clusters most likely originating from nested organization. The results are consistent with the Southern hybridization results, which classified the Bongo3 family as a highly abundant component of the *B. vulgaris* genome. The *Bingo*1-LTR probe produced signals that were predominantly located close to the pericentromeric regions of most chromosomes, although there were considerable differences in the strength of the signals between chromosomes (Figure 6C). The LTR probe of *Beon*1 hybridizes to only one chromosome pair (Figure 6D). Multicolor FISH on interphase and pachytene chromosomes indicated the interspersion of *Beon*1 copies (red fluorescence) in the 18S rRNA gene arrays (green fluorescence), which are located on chromosome 1 (Figure 6E, F). High-resolution fiber-FISH on extended genomic fibers revealed the genomic organization of rDNA arrays (green signals) within the genome of *B. vulgaris*. Two different types of rDNA subrepeats were identified: 1) those representing the canonical rDNA repeat without interruption by retrotransposon sequences and 2) those harboring *Beon*1 (Figure 6G). Both repeat types were found to be organized in interspersed sequence stretches.

**Figure 7:**
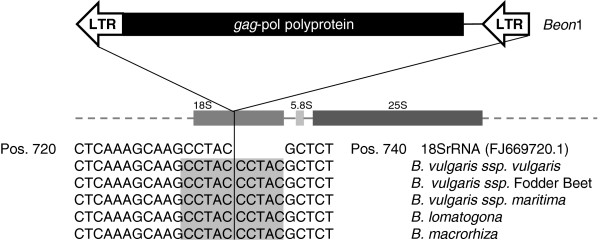
**The chromovirus *Beon*1 of the Galadriel clade is located exclusively within the 18S rRNA (ribosomal RNA) genes. **Members of the *Beon*1 family were found to be integrated at a conserved position of the 18S rRNA gene throughout species of different *Beta* sections; the integration in the opposite direction to the analyzed *Beon*1 copy within the *Beta vulgaris *bacterial artificial chromosome (BAC) is shown at the top. *Beon*1 integration sites were amplified from the following species of the section *Beta *(*B. vulgaris ssp. vulgaris *KWS2320 (cultivated beet) and *B. vulgaris ssp. maritima *(wild beet)), and wild beets of the section *Corollinae *(*B. lomatogona *and *B. macrorhiza)*. For the wild beet species *B. vulgaris ssp. maritima*, integration in the direction of rDNA transcription was confirmed by PCR. The wild beets *B. lomatogona *and *B. macrorhiza *harbor *Beon*1 copies, which were found to have an integration pattern in both orientations. The conserved target site duplication of *Beon*1 elements is indicated by grey boxes.

Sequence analysis of the BAC containing *Beon*1 and its flanking region confirmed the integration within the 18S rRNA gene (Figure 7). *Beon*1 integrated in the opposite direction to the rDNA transcription at nucleotide position 736, and exhibited a TSD 5 bp in length. In wild beets of the sections *Beta* and *Corollinae*, a similar integration pattern was identified by PCR using an 18S rRNA-specific primer and the LTR reverse primer of *Beon*1. This analysis identified Beon integrations in the direction of rDNA transcription in the wild beet *B. vulgaris ssp. maritima*, and in both orientations within the wild beet (*Corollinae*) genomes of *B. lomatogona* and *B. macrorhiza*.

### Transcriptional activity of *Beta* chromoviruses

More than 21,500 expressed sequence tags (ESTs; http://genomics.msu.edu/sugarbeet/blast.html) from sugar beet were screened for potential chromoviral transcripts. In general, chromoviruses seem to be barely expressed in the genome of *B. vulgaris*. Only 10 evaluable chromoviral transcripts were detected, which belonged to the CRM and Tekay clades. These ESTs had identity values greater than of 80% and were larger than 600 bp. Only four transcripts were associated with full-length chromoviral sequences; the others were generated from copies with internal deletions or they represent chimeric structures containing unrelated sequences. No ESTs were identified for *Beon*1 or for one of the *Bingo* families.

## Discussion

A large amount of genomic data has been generated by high-throughput sequencing technologies, which enable comparative analyses of retrotransposons within or across genomes. Short-read assemblies offer new possibilities for the detection of TEs; however, they are often excluded from assemblies because of their repetitiveness, genome-wide distribution, truncation, and nested organization [1,8,41,42].

We carried out a comparative analysis of chromoviruses in the beet genome, using data from a preliminary *B. vulgaris* genome draft sequence, which provides an insight into the genomic and chromosomal organization, distribution, and evolution of these viruses.

### Chromoviruses are widely distributed within genera of the Amaranthaceae family

Although the coding region of chromoviruses is relatively conserved within a clade, the sequence identity between the LTRs has been used for the grouping of the 22 chromoviruses into separate families [43]. In total, 16 different chromoviral families of the CRM, Reina, Tekay, and Galadriel clades have been identified, representing all known chromoviral clades in higher plants. Nevertheless, our analysis of genome-wide RT sequences clearly shows that more chromoviruses may be identified from the genome draft sequence, which is also evident from the signal strength in the blotting and *in situ* hybridization experiments. Only one similar, but less exhaustive analysis of chromoviruses has been conducted previously, which was for the *Musa acuminata* genome [44].

Our analysis shows that families from the same clade have conserved characteristics throughout the genera *Beta* and *Patellifolia*. In cultivated and wild beets, the *Bongo* families of the Tekay clade were the most abundant, followed by the *Beetle* families of the CRM clade. In comparison, *Beon*1 of the Galadriel clade most probably comprises a single family harboring only a few elements. Similarly, all *M. acuminata* Galadriel chromoviruses are members of a single family, designated Monkey [44]; however, Monkey members constitute about 0.2 to 0.5% of the genome [45]. In *M. acuminata*, the Reina clade constitutes more than half of all chromoviruses and makes up about 4% of the genome, followed by the Tekay clade, constituting about 2% [45].

### Chromosomal localization of *Beta* chromoviruses

The localization of plant chromoviruses on chromosomes has been investigated in several plants, focusing on members of the CRM clade that show a specific accumulation in centromeric regions. In banana, the chromoviruses of the Reina and Tekay clade were physically mapped in centromeric and peri-centromeric associated heterochromatin, whereas Monkey elements belonging to the Galadriel clade preferentially inserted into the NOR and co-localized with the rRNA genes [44]. This was also observed in this study for *Beon*1 in *B. vulgaris*, where the physical mapping indicated exclusive localization of *Beon*1 copies in the NOR. The results suggest that the localization might have been established by a single integration event of a *Beon*1 copy into the 18S rRNA gene of a common ancestor, as supported by the presence of *Beon*1 elements within the 18S rRNA genes of wild beets from the sections *Beta* and *Corolliflora*. Such integrations in the NOR region are not unusual for TEs, and have been reported for the long interspersed element (LINE) R2Bm of *Bombyx mori*[46]. Furthermore, *B. vulgaris* ribosomal RNA genes seem to tolerate TE integrations, as an insertion of a single BNR1 LINE has previously been reported [47]. Nevertheless, our FISH analysis clearly showed multiple *Beon*1 copies interrupting the 18S rRNA genes. The integration of several members of the tomato rDNA-related retrotransposon (TRRT) family within 18S rRNA genes was recently shown, and the existence of segmental duplication events rather than targeted integration has been proposed to explain retrotransposon amplification [48]. Our studies assigned TRRT elements to the Galadriel clade, forming a branch together with *Beon*1. The sequence conservation of *Beon*1 copies might result from strong purifying selection and homogenization of the coding sequences of the ribosomal DNA.

Integrations into genic regions were shown for families of the Reina and Tekay clade. A *Bongo*3 copy was identified in the vicinity of a disease resistance-activating factor [30], and the recently described chromovirus *Bert* (in this paper assigned to *Bingo* elements) was found within an intron of the callose synthase gene [49]. It is possible that these plant retrotransposons have the ability to alter the expression of nearby genes, as the importance of TEs for the epigenetic regulation of plant genomes has been stated previously [50-52].

### The functional role of the integrase

Several studies have confirmed the functional role of integrase for targeted integrations [13,14,53]. During the integration process, the interaction of DNA and integrase is crucial [54], and the relevance of the chromodomain in modulating the interaction with diverse chromatin components has already been shown [19,55]. An enrichment of positively charged amino acid residues was found within the integrase of all *Beta* chromoviruses. The differences in the extent of these residues possibly regulate the degree to which the integrase is capable of establishing electrostatic protein–protein interaction. This might enable the retrotransposon to sense target-specific chromatin states, as described by Roudier *et al*. [56]. Furthermore, we detected a potential NLS in all *Beetle* families of the CRM clade [57]. Such NLS signals are part of the integrase of several retroviruses and LTR retrotransposons, and are responsible for the transfer of the pre-integration complex to the nucleus [58-60].

The chromodomains of the plant chromoviral clades CRM, Reina, Tekay, and Galadriel in *B. vulgaris* are easily distinguishable by the presence or absence of conserved amino acid residues compared with HP1, and their position in relation to the *gag-pol* polyprotein. Although the chromodomain sequences have been classified into three groups [19,38], only group II chromoviruses have been identified in plants, with all of them belonging to the clades Tekay, Reina, and Galadriel.

The chromodomain encoded by CRM clade members such as *Beetle* extends into the 3^′^ LTR [21,35]. Because of this substantial difference from group II chromodomains, the *Beetle* chromodomains are referred to as CR motifs. A recent survey of CRM clade elements across diverse plant genomes assigned these chromoviruses to three different groups [40]. CRM chromoviruses of group A carry a CR motif, and are genuine centromeric retrotransposons, which probably transpose actively into centromeric regions. By contrast, group B members are not localized at the centromere, whereas group C representatives, despite a lack of the CR motif, were also found in centromeric regions. Interestingly, the *P. procumbens* chromovirus *Beetle*1 [21] and the *Beetle*7 chromovirus from *B. vulgaris* described in the current study share considerable amino acid identity (41%) within their chromodomain, and also have similar LTR lengths (1089 and 1086 bp, respectively), indicating a role for both *Beetle*1 and *Beetle*7 in the formation of functional centromeres. Amino acid conservations were also found within the chromodomains of CR elements from different grass species [40,61], supporting the assumption that the CR elements of grasses were derived from a single ancient family [62], and that conservation of the chromodomain is also crucial for centromere stability and thus host genome integrity.

### Transcriptional activity of *Beta* chromoviruses

Centromeres are thought to be determined epigenetically [63], including via a transcription-mediated mechanism [51,64]. Several RT-PCR studies have identified the transcriptional activity of chromoviruses, in particular of CRM clade members [21,40,64-66]. The analysis of *B. vulgaris* EST datasets indicates the capability of *Beetle* chromoviruses for autonomous transposition. Thus, the chromodomain as a key component of genuine CRs facilitates the targeting process into centromeric regions, and might be therefore responsible for the generation of centromeric transcripts, which are involved in RNA interference-mediated centromere identity and function.

The rRNA genes have high transcriptional activity, thus it is possible that read-through transcripts of *Beon*1 might be generated. Alternatively, as the *Beon*1 copies harbor intact coding sequences, their reverse transcription and the integration of new copies into the genome is conceivable. However, corresponding transcripts were not detected in the EST database. Hence, epigenetic silencing mechanisms might prevent the reverse transcription and spreading of *Beon*1 copies into other chromosomal regions. This could be caused by the insertion of *Beon*1 in two orientations, as was shown in wild beet species, whereas transcription would result in double-stranded RNA, which would immediately initiate the RNA interference machinery. Subsequently, Dicer-generated small interfering RNAs would serve as substrates in RNA-induced transcriptional silencing (RITS) complexes or RNA-induced silencing complex (RISC). RITS would initiate the transcriptional silencing of *Beon*1 copies by RNA-directed DNA methylation. Most likely, is the post-transcriptional silencing of *Beon*1 copies ed by degradation of mRNA mediated by RISC. In plants, it has been shown that rDNA transcription is subject to dosage control [67], with only a subset of rDNA genes being transcribed. It might be possible therefore that rDNA genes containing *Beon*1 copies are not transcribed.

Based on the accumulation of mutations within their LTRs [2], we calculated the age of the transposition events and concluded that members of the 16 families transposed less than two million years ago. Therefore, these transpositions are evolutionarily recent events. However, families of the four clades are likely to be much older, as deduced from their widespread distributions within the genera *Beta* and *Patellifolia*. Furthermore, nearly 70% of the analyzed contigs of the draft sequence of the beet genome contain incomplete or recombined copies, which over time have lost the typical retrotransposon hallmarks. Ma *et al.*[41] found that LTR retrotransposons are subject to a genome-specific recombination rate that results in a half-life of less than 6 million years in rice or 3 million years in *Arabidopsis*[43].

## Conclusions

Chromoviruses have been shown to make up a large fraction of LTR retrotransposons in the genome of *B. vulgaris* and related wild beets. In our study, we focused on complete members of all four chromoviral clades known in higher plants. Intact members could still be capable of autonomous transposition, which is in agreement with the relatively recent transposition for single elements such as *Bingo*1, *Bongo*3, and *Beetle*7. Based on representative members of the four plant chromoviral clades, we were able to show their widespread presence within the family of Amaranthaceae, indicating their ancient origin. The analysis of the chromodomain-containing Ty3-*gypsy* retrotransposons provides valuable information for the annotation of the repeated DNA fraction of *Beta* genomes and is important for the understanding of the contribution of the chromodomain to retrotransposon guidance.

## Methods

### Plant material and preparation of genomic DNA

Plants of *B. vulgaris* ssp. *vulgaris* KWS2320, *B. patula* (BETA 548), *B. corolliflora* (BETA 846), *B. nana* (BETA 541), *P. procumbens* (BETA 951), and *S. oleracea* ‘Matador’ were grown under greenhouse conditions. Wild beet seeds were obtained from Dr L Frese (Julius Kühn Institute (JKI), Federal Centre for Breeding Research on Cultivated Plants, Quedlinburg, Germany, and from the Genbank of the Plant Genome Resources Center, Gatersleben, Germany), and genomic DNA was isolated from the young leaves using the cetyltrimethyl ammonium bromide (CTAB) protocol [68].

### Computational methods

A local database of a preliminary *B. vulgaris* genome assembly was queried using clade-specific chromovirus *gag-pol* polyproteins. The unedited and non-public assembly RefBeet-0.1.1 draft comprises 628 Mb in 346,000 contigs with an N50 contig size of approximately 4000 bp (a current version of the sugar-beet genome draft (RefBeet-0.9) is available for download at http://bvseq.molgen.mpg.de). Additionally, for the *in silico* identification of chromoviral sequences, 20.4 Mbp of BAC end-sequence data from US H20 clones [69] were used.

For the identification of chromoviral RTs, an HMM-based strategy was used with *hmmbuild* and *hmmsearch* programs of the *HMMER3* package (hmmer.janelia.org, [70]). Based on a Ty3-*gypsy* RT alignment containing 96 references of diverse eukaryotes (described by Llorens *et al*. [71], and downloadable from http://www.gydb.org[72]), an HMM specific for Ty3-*gypsy* RTs was constructed and used to query a computationally translated *B. vulgaris* genome assembly. Hits with a bit score greater than 50 and a length of more than 200 amino acids were selected, aligned using the MUSCLE algorithm [73] and compared with the previously published chromoviral references [71]. All hits that could be assigned to a chromoviral clade were kept for further analysis.

Phylogenetic analyses were conducted using *MEGA* version 4 [74]. The constructed neighbor-joining tree of the ClustalW alignment of the complete *gag-pol* polyprotein sequences was calculated, using bootstrap values of 1000 cycles.

Integration times were calculated using the equation t = K/2r, where t is the age, K is the number of nucleotide substitutions per site between each LTR pair, and r is the nucleotide substitution rate. An average synonymous substitution rate of 1.5 × 10^-8^ mutations/site/ year as determined for the chalcone synthase and *Adh* loci in *A. thaliana*[75] was used.

### PCR analyses, cloning, and sequencing

The amplification of different retrotransposon regions for hybridization experiments was carried out using the primers listed in Table 2. PCR from genomic or BAC DNA (isolated with Nucleo Bond BAC 100 kit; Macherey-Nagel GmbH & Co. KG, Düren, Germany) were performed (Expand High Fidelity PCR system; Roche Diagnostics GmbH, Mannheim, Germany) at 94°C for 3 minutes, 35 cycles at 94°C for 30 seconds, 52 to 56°C for 30 seconds, and 72°C for 30 to 60 seconds, with a final extension at 72°C for 5 minutes. The suppression PCR method [29] was used to amplify the LTR sequences of the retrotransposon *Beon*1 utilizing the outward-facing primers *Beon*1-PBS, starting from the primer binding site, and *Beon*1-PPT, starting from the polypurine tract, shown in Table 2 and. A prolonged elongation time of 6 minutes was used for the amplification of *Beon*1 sequences from adaptor-ligated BAC DNA. For amplification of the *Beon*1 integration site, 18S rRNA-specific primers were used. Amplicons were cloned into a cloning vector (pGEM-T; Promega Corp. Madison, WI, USA) and transformed into *Escherichia coli* DH10B cells (Stratagene, La Jolla, CA, USA) by electroporation. Clones were sequenced in an automated capillary sequencing system (CEQ 8000; Beckman Coulter Inc., Brea, CA, USA) using M13 universal or sequence-specific primers. Raw sequence data were analyzed with DNASTAR software (DNASTAR Inc., Madison, WI, USA). 

**Table 2:** Primers for amplification of specific chromoviral domains, LTR sequences and integration sites

**Amplified domain**	**Probe number**	**Forward primer**	**Reverse primer**	**Length (bp)**
*Beetle*7 LTR	1	TAACTAGCTCGGTTTGTCC	CACTCCCCTAAGTCTTTCC	296
*Beetle*3 RT	2	AGGGGATGAATGGAAGACG	GAAACTAATCTCTCCTTGC	289
*Bongo*3 LTR	3	ATTTGCTTGTATGCTACATG	TTATTGTCCAACTCTAGACG	467
*Bongo*3 RT	4	AAGAACAAGTACCCTTTGCC	GAGAACTTGGCATATAATTG	368
*Bingo*1 LTR	5	AAGGGCAACGGTCACTGGC	TTACAAGATAGGTTCAAGGC	246
*Bingo*1 RT	6	CGAAGATGCTCACAAAACGGC	GCACTATACACAAGAATATCG	177
*Beon*1 LTR	7	CACAAACATGAGATGGTGC	CGGACTACTAGACAAGGC	373
*Beon*1 RT	8	ATGAATAAAATCTTCCAGCC	CCTAAGAAAGACACCTCG	191

Abbreviations: LTR, long terminal repeat; PBS, primer binding site; PPT, polypurine tractRT, reverse transcriptase.

### Southern hybridization

For gel electrophoresis, genomic DNA was digested with the endonuclease *Hin*dIII and separated in 1.2% agarose gels, then transferred onto positively charged nylon membranes (GE Healthcare, Princeton, NJ, USA) using alkaline transfer. Southern hybridizations using ^32^P-labeled probes were performed in accordance with standard protocols [76]. Filters were hybridized at 60°C overnight, then washed at 60°C in 2 × SSC/0.1% SDS and 1 × SSC/0.1% SDS for 10 minutes each. Signals were detected by autoradiography.

### Fluorescence *in situ* hybridization

The meristem of young leaves was used for the preparation of mitotic chromosomes. Before fixation in methanol:acetic acid (3:1), leaves were incubated for 4 hours in 2 mmol/l 8-hydroxyquinoline. Fixed plant material was macerated in an enzyme mixture consisting of 0.3% (w/v) cytohelicase, 1.8% (w/v) cellulase from *Aspergillus niger* (Sigma-Aldrich Chemie GmbH, Taufkirchen, Germany), 0.2% (w/v) cellulase Onozuka-R10 (SERVA Electrophoresis GmbH, Heidelberg, Germany) and 20% (v/v) pectinase from *A. niger*, (Sigma-Aldrich Chemie GmbH) followed by dropping the nuclei suspension onto slides as described previously [77]. FISH was performed on *B. vulgaris* chromosomes in accordance with the protocol of Heslop-Harrison *et al*. [78], modified for beet by Schmidt *et al*. [79]. For high-resolution FISH, the chromatin fibers were prepared as described previously [80]. The LTR fragments of *Beetle*7, *Bongo*3, *Bingo*1, *Beon*1 and the 18S rRNA-specific clone pZR18S [GenBank: HE578879] were used as probes after labeling by PCR in the presence of biotin-11-dUTP or digoxigenin-11-dUTP. Chromosome preparations were counterstained with 4^′^,6^′^-diamidino-2-phenylindole (DAPI) and mounted in antifade solution (CitiFluor Ltd, Leicester, UK). Slides were examined under a fluorescence microscope (Axioplan 2 imaging; Carl Zeiss, Jena, Germany equipped with filter numbers 02 (DAPI) ,09 (FITC), and15 (Cy3). Images were acquired directly with Applied Spectral Imaging software (v.3.3; Applied Spectral Imaging, Carlsbad, CA, USA) coupled with the high-resolution CCD camera ASI BV300-20A.

### Sequence accessions

The accession numbers of all chromoviruses analyzed in this article are listed in Table 3. Additionally, the accession numbers of the HP1 proteins of *Drosophila melanogaster* (Dm-HP1) and *Arabidopsis thaliana* (At-LHP1) used for the analysis of the chromodomain are included.

**Table 3:** Accession numbers of analyzed chromoviruses and reference HP1 proteins

**Sequence**	**Organism**	**GenBank accession number**
*Beetle*1	*Patellifolia patellaris*	AJ539424
*Beetle*2	*P. patellaris*	FM242082
*Beetle*3	*Beta vulgaris*	JX455076
*Beetle*3-1	*B. vulgaris*	JX455077
*Beetle*4	*B. vulgaris*	JX455078
*Beetle*4-1	*B. vulgaris*	JX455079
*Beetle*4-2	*B. vulgaris*	JX455080
*Beetle*5	*B. vulgaris*	JX455081
*Beetle*5-1	*B. vulgaris*	JX455082
*Beetle*5-2	*B. vulgaris*	JX455083
*Beetle*6	*B. vulgaris*	JX455084
*Beetle*7	*B. vulgaris*	JX455085
*Bongo*1	*B. vulgaris*	JX455095
*Bongo*2	*B. vulgaris*	JX455096
*Bongo*3	*B. vulgaris*	EF101866
*Bingo*1	*B. vulgaris*	JX455086
*Bingo*1-1	*B. vulgaris*	JX455087
*Bingo*2	*B. vulgaris*	JX455088
*Bingo*3	*B. vulgaris*	JX455089
*Bingo*4	*B. vulgaris*	JX455090
*Bingo*5	*B. vulgaris*	JX455091
*Bingo*6	*B. vulgaris*	JX455092
*Bingo*7	*B. vulgaris*	JX455093
*Beon*1	*B. vulgaris*	JX455094
CRM2	*Zea mays*	AC129008
Tekay	*Z. mays*	AF050455
Reina	*Z. mays*	ZMU69258
Galadriel	*Lycopersicon esculentum*	AF119040
Maggy	*Magnaporthe grisea*	L35053
Pyret	*M. grisea*	AB062507
Sushi-ichi	*Takifugu rubripes*	AF030881
PpatensLTR1	*Physcomitrella patens*	XM_001752430
TRRT1	*Solanum lycopersicum*	AC215351
Dm-HP1^a^	*Drosophila melanogaster*	M57574
At-LHP1^a^	*Arabidopsis thaliana*	AAL04059

Abbreviations: HP, heterochromatin protein; LHP, Like heterochromatin protein (*Arabidopsis *homolog).

^a^HP1 proteins.

## Abbreviations

BAC: Bacterial artificial chromosome; CR: Centromeric retrotransposon; DAPI: 4^′^ 6^′^-diamidino-2-phenylindole; FISH: Fluorescence *in situ* hybridization; FITC: Fluorescein isothiocyanate; HMM: Hidden Markov Model; HP1: heterochromatin protein1; K: Arginine; LHP: Like heterochromatin protein (*Arabidopsis* homolog); LTR: Long terminal repeat; NLS: Nuclear localization signal; NOR: Nucleolus organizer region; ORF: Open reading frame; P: Proline; PBS: Primer binding site; PPT: Polypurine tract; R: Lysine; RT: Reverse transcriptase; SDS: Sodium dodecyl sulphate; SSC: Saline sodium citrate; TAE: Standard tris acetate buffer; TE: Transposable element; TSD: Target site duplication; UTR: Untranslated region; W: Tryptophan; Y: Tyrosine.

## Competing interests

The authors declare that they have no competing interests.

## Authors’ contributions

The project was designed by B Weber and co-ordinated by TS. TH performed the bio-informatic analyses shown in Figure 3. DH, B Weisshaar, AEM, JCD, and HH provided the unpublished sugar beet genome draft. B Weber wrote the manuscript, and TH and TS contributed to the writing. All authors have read and approved the final manuscript.

## Supplementary Material

Additional file 1 **Primer binding sites used for reverse transcription by chromoviruses of *B. vulgaris.*** Alignment of chromoviral primer binding sites (PBSs) complementary to the initiator tRNA of methionine (tRNA_i_^Met^) and polypurine tracts (PPTs). The most abundant nucleotides are highlighted in black. Click here for file

Additional file 2 **The chromoviruses of *B. vulgaris *contain different chromointegrases. **The alignment was produced using the MUSCLE algorithm [74]. The shading marks a 50% consensus, with the black and grey boxes indicating identical and similar amino acid residues, respectively. Conserved motifs were identified at positions 1 to 41 (zinc finger), 132 to 168 (D, D_35_E), and 311 to 313 (GPF/GPY). The predicted start of the chromodomain is located at position 377 of the alignment. Click here for file

Additional file 3 **Blot gels corresponding to Figure 5. **The genomic DNA indicated above was digested with Hin*dIII *and separated in four equally loaded parts onto a single 1.2% agarose gel. Click here for file
