# Supplementary material for: Highly diverse chromoviruses of Beta vulgaris are classified by chromodomains and chromosomal integration
Source: Mob DNA. 2013 Mar 1;4:8. doi: 10.1186/1759-8753-4-8 (PMC3605345; doi:10.1186/1759-8753-4-8)
Supplement: Additional file 1 — Primer binding sites used for reverse transcription by chromoviruses of B. vulgaris. Alignment of chromoviral primer binding sites (PBSs) complementary to the initiator tRNA of methionine (tRNAiMet) and polypurine tracts (PPTs). The most abundant nucleotides are highlighted in black. [file 1759-8753-4-8-S1.pdf]

```
                                 PBS                                      PPT

Beetle3     CA  (AT)   T-GGTATCA-GAGCAAAGGTGT....//....   AAGAAGGAGGGAA   TG
Beetle3-1   CA  (AT)   T-GGTATAA-GAGCAAAGGCGT....//....   AAGAAGGAGGGAA   TG
Beetle4     CA  (TCT)  T-GGTATCA-TAGCTACAACAA....//....  AAAAAGGGGAGGA-   TG
Beetle4-1   CA  (TCT)  T-GGTATCA-TAGCTACAACAA....//....  AAAAAGGGGAGGA-   TG
Beetle4-2   CA  (TCT)  T-GGTATCA-GAGCTACAGCAA....//....  AAGAAGGGGAGGA-   TG
Beetle5     CA  (TA)   T-GGTATCAAGAGCATAGGTGA....//....AAAGAAGAGGAGGA-   TG
Beetle5-1   CA  (TC)   T-GGTATCAAGAGCTTTGGTGA....//....AAAGAAGAGGAGGA-   TG
Beetle5-2   CA  (TC)   T-GGTATCAAGAGCTTTGGTGA....//....AAAGAAGAGGAGGA-   TG
Beetle6     CA  (AG)   T-GGTATCA-GAGCTCTAGGTT....//....GAAGAAAGGGGGGA-   TG
Beetle7     CA  (TT)   T-GGTATCA-GAGCTTAAGGTT....//....GAACCTGAGGAGAA-   TG

Bongo1      CA  (GT)   T-GGTATCA-GAGCAAAATGTT....//....   AGGGAGGGGAGAT   TG
Bongo2      CA  (GT)   T-GGTATCA-GAGCAAAACGTT....//....   AAGGAGGGGAGAT   TG
Bongo3      CA  (AAG)  T-GGTATCT-GGGAGCTTTAGG....//....   AAGGGGGGTAGAC   TG

Bingo1      CA  (ACT)  T-GGTATCA-GAGCAGTAAGAT....//....   GGGCCCGGGGTAA   TG
Bingo1-1    TA  (ACT)  T-GGTATCA-GAGCAGTAAGAT....//....   GGGCCCGGGGTAA   TG
Bingo2      CA         TAGGTATCA-GAGCAGTTGAGT....//....   AAGGGAAGGGCAT   TG
Bingo3      CA  (TCT)  T-GGTATCA-GAGCACGTTGAT....//....    GGCCCGGGGTAA   TG
Bingo4      CA  (ACT)  T-GGTATCA-GAGCAAGGTTGC....//....   GGGACCAGGGTAA   TG
Bingo5      CA  (T)    T-GGTATCA-GAGCCAACCGCT....//....AAAGGGGGGAAGTTA   TG
Bingo6      CA  (T)    T-GGTATCA-GAGCATAACCTG....//....   AAGGGAGGAGGAA   TG
Bingo7      CA  (T)    T-GGTATCA-GAGCAGAACGAC....//....   AAGGAGGGAGTAT   TG

Beon1       CA  (CG)   T-GGTATCA-GAGCAGCAACGG....//....    AGTGGGGGAGAA   TG
tRNAi^Met              A-CCAUAGU-CUCGAAA
```

Additional file 1 Figure 1

Weber et al.
