# Supplementary material for: Highly diverse chromoviruses of Beta vulgaris are classified by chromodomains and chromosomal integration
Source: Mob DNA. 2013 Mar 1;4:8. doi: 10.1186/1759-8753-4-8 (PMC3605345; doi:10.1186/1759-8753-4-8)
Supplement: Additional file 2 — The chromoviruses of B. vulgaris contain different chromointegrases. The alignment was produced using the MUSCLE algorithm [74]. The shading marks a 50% consensus, with the black and grey boxes indicating identical and similar amino acid residues, respectively. Conserved motifs were identified at positions 1 to 41 (zinc finger), 132 to 168 (D, D35E), and 311 to 313 (GPF/GPY). The predicted start of the chromodomain is located at position 377 of the alignment. [file 1759-8753-4-8-S2.pdf]

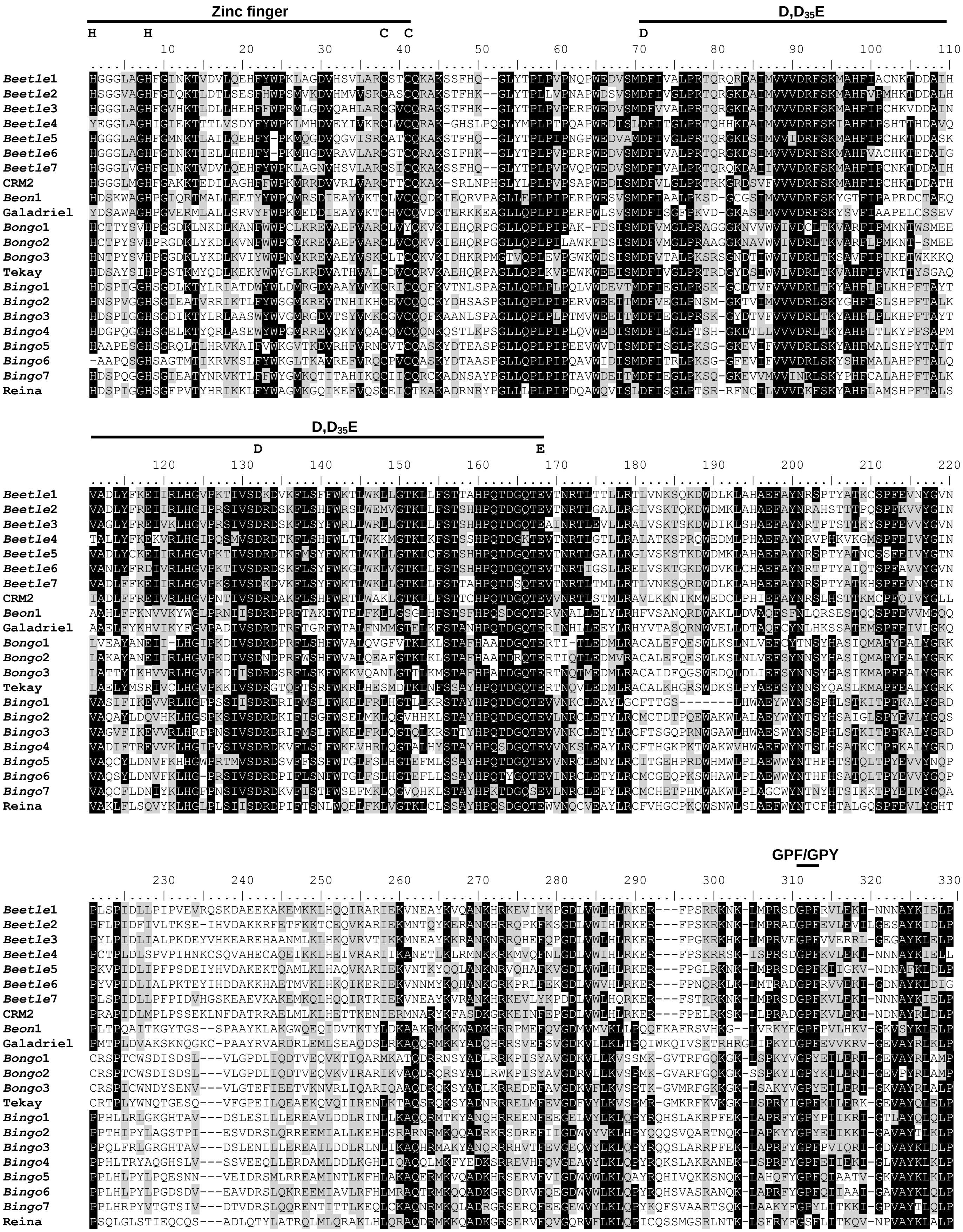
Zinc finger
D,D35E
GPF/GPY
Beetle1
Beetle2
Beetle3
Beetle4
Beetle5
Beetle6
Beetle7
CRM2
Beon1
Galadriel
Bongo1
Bongo2
Bongo3
Tekay
Bingo1
Bingo2
Bingo3
Bingo4
Bingo5
Bingo6
Bingo7
Reina

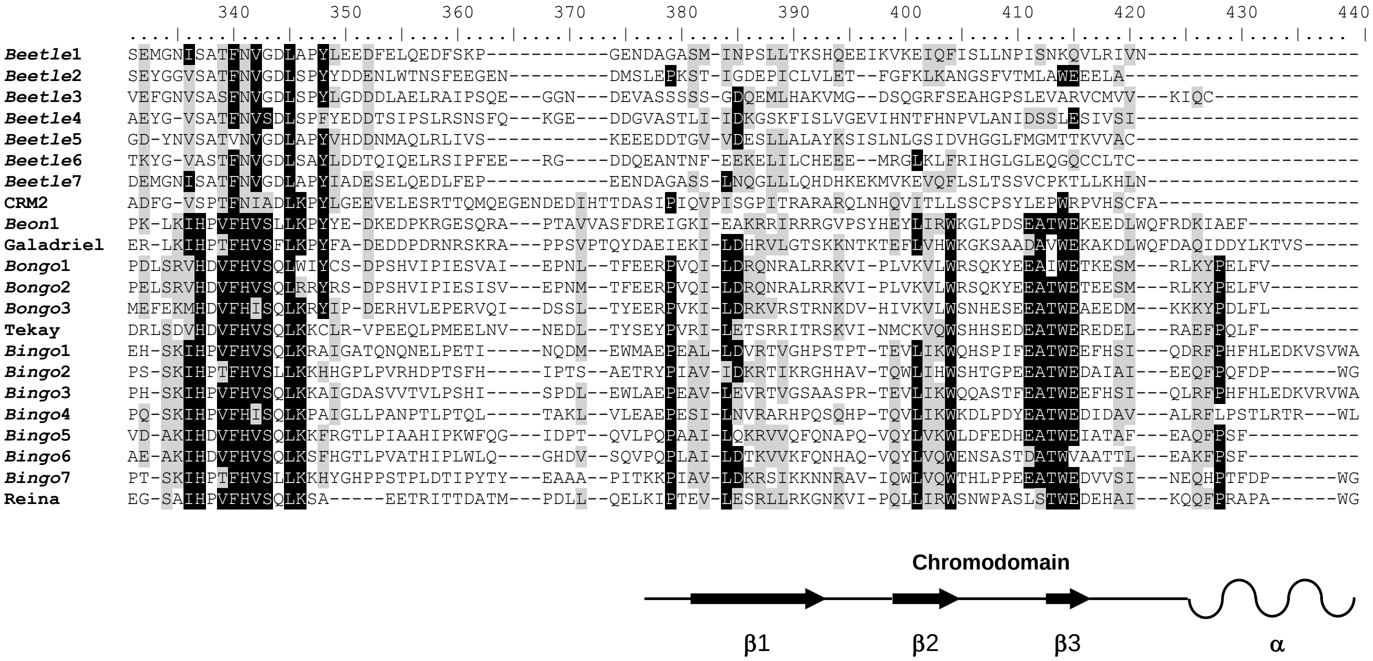

Additional file 2 Figure 2

Weber et al.
