# Supplementary figures and images for: Highly diverse chromoviruses of Beta vulgaris are classified by chromodomains and chromosomal integration
Source: Mob DNA. 2013 Mar 1;4:8. doi: 10.1186/1759-8753-4-8 (PMC3605345; doi:10.1186/1759-8753-4-8)

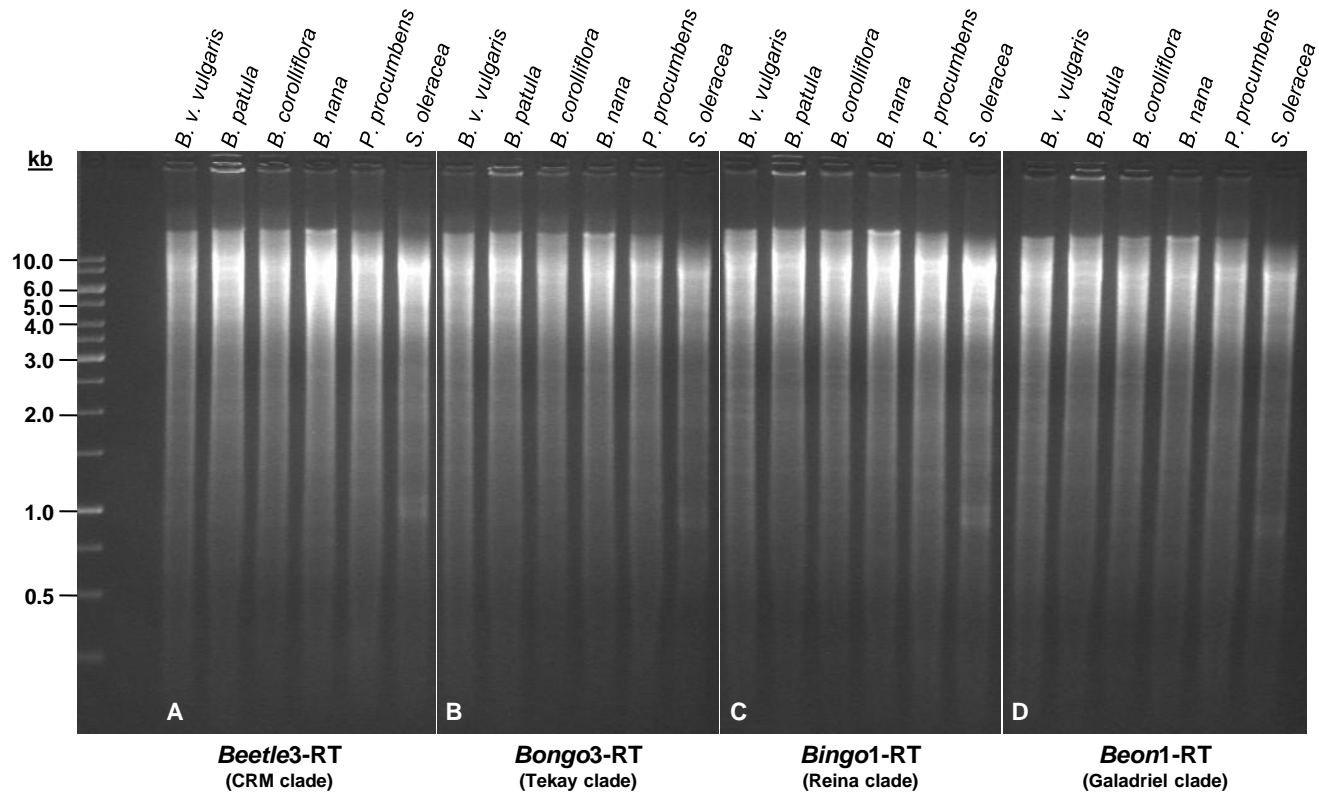

**Additional file 3 Figure 3**  
**Weber et al.**

Supplement: Additional file 3 — Blot gels corresponding to Figure 5. The genomic DNA indicated above was digested with HindIII and separated in four equally loaded parts onto a single 1.2% agarose gel. [file 1759-8753-4-8-S3.pdf]
